# Intermittent Hypoxia Influences Alveolar Bone Proper Microstructure via Hypoxia-Inducible Factor and VEGF Expression in Periodontal Ligaments of Growing Rats

**DOI:** 10.3389/fphys.2016.00416

**Published:** 2016-09-16

**Authors:** Shuji Oishi, Yasuhiro Shimizu, Jun Hosomichi, Yoichiro Kuma, Hideyuki Maeda, Hisashi Nagai, Risa Usumi-Fujita, Sawa Kaneko, Naoki Shibutani, Jun-ichi Suzuki, Ken-ichi Yoshida, Takashi Ono

**Affiliations:** ^1^Department of Orthodontic Science, Graduate School of Medical and Dental Sciences, Tokyo Medical and Dental UniversityTokyo, Japan; ^2^Department of Forensic Medicine, Graduate School of Medicine, Tokyo Medical UniversityTokyo, Japan; ^3^Department of Legal Medicine (Forensic Medicine), Keio University School of MedicineTokyo, Japan; ^4^Department of Advanced Clinical Science and Therapeutics, The University of TokyoTokyo, Japan

**Keywords:** bone mineral density, hypoxia inducible factor, intermittent hypoxia, periodontal ligament, vascular endothelial growth factor

## Abstract

Intermittent hypoxia (IH) recapitulates morphological changes in the maxillofacial bones in children with obstructive sleep apnea (OSA). Recently, we found that IH increased bone mineral density (BMD) in the inter-radicular alveolar bone (reflecting enhanced osteogenesis) in the mandibular first molar (M1) region in the growing rats, but the underlying mechanism remains unknown. In this study, we focused on the hypoxia-inducible factor (HIF) pathway to assess the effect of IH by testing the null hypothesis of no significant differences in the mRNA-expression levels of relevant factors associated with the HIF pathway, between control rats and growing rats with IH. To test the null hypothesis, we investigated how IH enhances mandibular osteogenesis in the alveolar bone proper with respect to HIF-1α and vascular endothelial growth factor (VEGF) in periodontal ligament (PDL) tissues. Seven-week-old male Sprague–Dawley rats were exposed to IH for 3 weeks. The microstructure and BMD in the alveolar bone proper of the distal root of the mandibular M1 were evaluated using micro-computed tomography (micro-CT). Expression of HIF-1α and VEGF mRNA in PDL tissues were measured, whereas osteogenesis was evaluated by measuring mRNA levels for alkaline phosphatase (ALP) and bone morphogenetic protein-2 (BMP-2). The null hypothesis was rejected: we found an increase in the expression of all of these markers after IH exposure. The results provided the first indication that IH enhanced osteogenesis of the mandibular M1 region in association with PDL angiogenesis during growth via HIF-1α in an animal model.

## Introduction

Intermittent hypoxia (IH) during sleep has been implicated in the pathogenesis of obstructive sleep apnea (OSA; Noda et al., 1998; Lal et al., 2012), although the role of IH in the growth of children with OSA has not been clarified. In particular, it has been reported that pediatric OSA is frequently associated with impairment in the growth and development of craniofacial and otolaryngological tissues, as well as with neuromuscular diseases (Balbani et al., 2005; Huang and Guilleminault, 2012). Hypertension, cardiac remodeling, and other complications of OSA have been studied using rodent models of IH induced by short cycles of hypoxia–normoxia (Skelly et al., 2012; Maeda et al., 2013; Nagai et al., 2015). Previously, we demonstrated that IH exposure induces a decrease in the volume of the nasal cavity, reflecting impaired development of maxillofacial bones (Kuma et al., 2014). Other researchers showed that IH increased the bone mineral density (BMD) in the inter-radicular alveolar bone in the mandibular first molar (M1) region during growth (Oishi et al., 2015), but the underlying mechanism remains unknown.

Hypoxia is critical to the remodeling and repair of damaged bones via hypoxia-inducible factor (HIF; Maes et al., 2012), the key stimulator of vessel formation and angiogenesis. The gene encoding the prominent angiogenic factor vascular endothelial growth factor (VEGF) is the primary target for HIF-1α (Riddle et al., 2009). A recent study provided evidence supporting the view that HIFs and VEGF play essential roles in coupling between angiogenesis and osteogenesis during bone formation and repair (Schipani et al., 2009).

Alveolar bone proper is covered with collagen fibers in the periodontal ligament (PDL; Shimizu et al., 2014). The PDL is a specialized soft connective tissue that connects the tooth with the alveolar bone socket, thereby promoting the development and maintenance of periodontium (Kaku and Yamauchi, 2014). PDL tissues are comprised of PDL cells (Shimizu et al., 2014), collagen fibers (Kaku and Yamauchi, 2014), blood vessels (Muramoto et al., 2000), nerve elements (Muramoto et al., 2000), extracellular substances (Kaneko et al., 2001), osteoclasts (Kaneko et al., 2001), and osteoblasts (Mayahara et al., 2012), and provides progenitor cells for bone formation and remodeling. Alkaline phosphatase (ALP) and bone morphogenetic protein-2 (BMP-2) are known to induce osteogenesis and the osteogenic transformation of PDL cells (Kuru et al., 1999; Selvig et al., 2002). ALP activity reflects early osteogenic differentiation in the presence of osteoblasts (Kuru et al., 1999). Previous data showed that ALP activation and BMP-2 upregulation in PDL cells induce periodontium osteogenesis in response to growth hormones (Li et al., 2001) or matrix Gla proteins (Li et al., 2012). In addition, mechanical forces such as moderate occlusal stimuli and dissipation of masticatory force are transmitted from the teeth through the PDL to the progenitor cells, thereby promoting bone remodeling in the periodontal tissue (Chen et al., 2005). As such, PDL tissues maintain proper alveolar bone homeostasis via ALP and BMP-2.

Bone remodeling, a complex process by which old bone is continuously replaced by new tissue, is affected by a variety of biochemical and mechanical factors (Hadjidakis and Androulakis, 2006). With regard to signaling pathways, the Wnt (Wang et al., 2014), OPG/RANKL/RANK (Hsu et al., 2006), and HIF pathways (Mamalis and Cochran, 2011) are well-known in the control of bone remodeling. In this study, we specifically focused on the HIF pathway to assess the effect of IH by testing the null hypothesis that no significant differences in the mRNA-expression levels of relevant factors associated with the HIF pathway in PDL occur between control rats and growing rats with IH. To this end, we analyzed the microarchitecture and mineral density of the alveolar bone proper around the mandibular M1 after 3 weeks of IH in growing rats, with reference to HIF1-α, VEGF, ALP, and BMP-2 mRNA expression.

## Materials and methods

### Experimental IH model

Experiments were conducted on twelve 7-week-old male Sprague–Dawley rats randomly divided into two groups. Experimental rats were exposed to IH at a rate of 20 cycles per h (nadir, 4% oxygen; peak, 21% oxygen; 0% carbon dioxide) for 3 weeks (IH group), and control rats breathed room air (C group). The control cage was placed next to the cage equipped with the IH apparatus, and all rats underwent their respective treatments for 8 h per day during the 12-h “lights on” period (Maeda et al., 2013; Nagai et al., 2015). The experiments were conducted while the rats were 7 to 10 weeks of age, when craniofacial bones actively develop (puberty), as documented by studies of craniofacial growth (Spence, 1940) and puberty onset (Cheung et al., 2001) in male rats. All rats were allowed free access to food and water throughout the experimental period, as previously described (Skelly et al., 2012; Maeda et al., 2013; Nagai et al., 2015). Immediately after the IH-exposure period, all rats were anesthetized by a sodium pentobarbital injection and sacrificed. All experimental procedures were performed according to the Guide for the Care and Use of Laboratory Animals published by the US National Institutes of Health (NIH publication 85-23, revised 1996). The animal protocol was approved by the Animal Experimental Committee of Tokyo Medical University (approval number S-26063).

### Three-dimensional microcomputed tomography (Micro-CT) analysis

**Figure 1:**
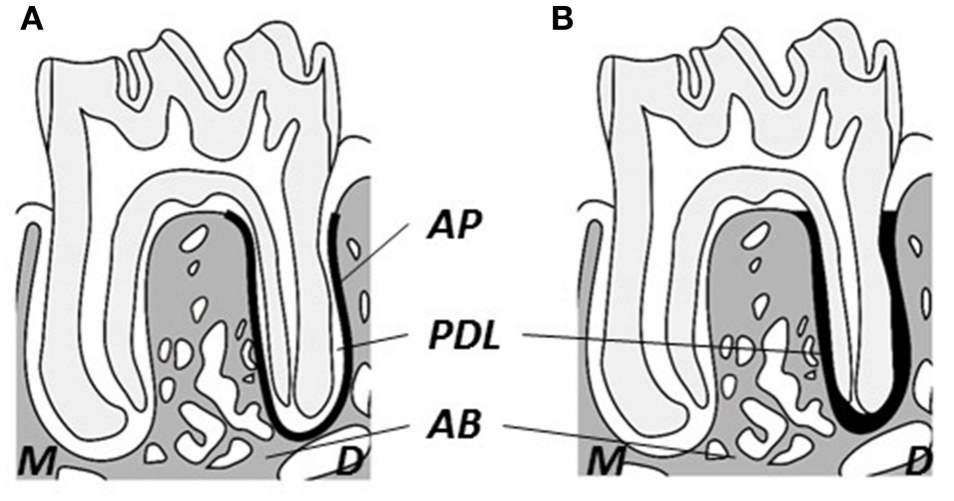
**Schematic drawing of observational area of micro-CT. (A)** Observational area for alveolar bone proper (heavy black line). **(B)** Observational area for the periodontal ligament (heavy black area). M, Mesial; D, distal; AB, inter-radicular alveolar bone; AP, alveolar bone proper; PDL, periodontal ligament.

Changes in the bony microstructure of the alveolar bone proper around roots of the mandibular M1 and in the PDL space were investigated using micro-CT with a desktop X-ray micro-CT system (SMX-100CT; Shimadzu, Kyoto, Japan) with a scanning resolution of 20-μm intervals on individual images. The region of interest (ROI) for structural morphometry was enlarged to a 40-μm radius around the distal root (Figure 1A; Shimizu et al., 2014). Each ROI was analyzed with respect to BMD, bone volume/tissue volume (BV/TV), trabecular thickness (Tb.Th), and trabecular number (Tb.N) using three-dimensional image-analysis software (TRI/3D-BON; Ratoc System Engineering, Tokyo, Japan). Additionally, to evaluate changes from mechanical stimuli in the PDL space, tissue volume (TV) around the distal root of the mandibular M1 was computed (Figure 1B; Shimizu et al., 2014).

### Reverse transcription quantitative real-time PCR (RT-qPCR) analysis

PDL tissues were removed from extracted roots of the mandibular M1. Total RNA was isolated from PDL tissues using the PureLink^TM^ FFPE Total RNA Isolation Kit (Invitrogen, CA, USA) according to instructions provided by the manufacturer (Afanador et al., 2005; Luan et al., 2007). cDNA was synthesized from total RNA with reverse transcription random primers using High-Capacity cDNA Reverse Transcription Kits (Applied Biosystems, Foster City, CA, USA). Quantitative PCR assays were carried out in triplicate for each sample using a 7500 Real-Time PCR System (Applied Biosystems, Foster City, CA, USA). PCR analyses were conducted with gene-specific primers and fluorescently labeled TaqMan probes (Takara Bio, Shiga, Japan). Appropriate primers were chosen for real-time PCR amplification of VEGF (forward primer: 5′-ccttgctgctctacctccac-3′, reverse primer: 5′-ccacttcgtgatgattctgc-3′), HIF-1α (forward primer: 5′-ctaccagaagggcaggatacag-3′, reverse primer: 5′-gca ggcagatgaaataccagtc-3′), ALP (forward primer: 5′-acgtggctaagaatgtcatc-3′, reverse primer: 5′-ctggtaggcgatgtcctta-3′), BMP-2 (forward primer: 5′-tcaagccaaacacaaacagc-3′, reverse primer: 5′-acgtctgaacaatggcatga-3′), and Hprt-1 (forward primer: 5′-cagactttgctttccttgg-3′, reverse primer: 5′-tccactttcgctgatgacac-3′). The thermocycling conditions used were 95°C for 30 s, followed by 40 cycles of 95°C for 5 s and 60°C for 34 s. Gene expression levels were calculated according to the ΔΔCT method of relative quantification. The threshold cycle (Ct) value of the target mRNAs (VEGF, HIF1α, ALP, or BMP-2) was normalized to the Ct values of the internal control (Hprt-1) in the same sample (ΔCt = Ct_target_ – Ct_Hprt−1_), followed by normalization to the control (ΔΔCt = ΔCt_IHgroup_ – ΔCt_Cgroup_). The fold change in expression was calculated as the relative quantification value (RQ; 2^−ΔΔCt^; Livak and Schmittgen, 2001).

### Statistical analysis

Statistical calculations were performed using statistical analysis software (IBM SPSS Statistics Version 20.0 Chicago, IL, USA). We first examined the normality and variance of the data using the *F*-test. The control and experimental groups were compared using the Mann–Whitney *U*-test for nonparametric data, and statistical significance was established at a *p* level of less than 0.05.

## Results

### Body weight changes in rats after IH

The median body weight (mean ± standard error) of rats exposed to IH for 3 weeks (277.5 ± 3.8 g) was significantly lower than that of control (C) rats (356.0 ± 9.8 g). Previous reports using this animal model showed that the correlation between the body weight and whole-body growth in this IH model was low (Maeda et al., 2013; Kuma et al., 2014; Oishi et al., 2015).

### Three-dimensional Micro-CT analysis

**Figure 2:**
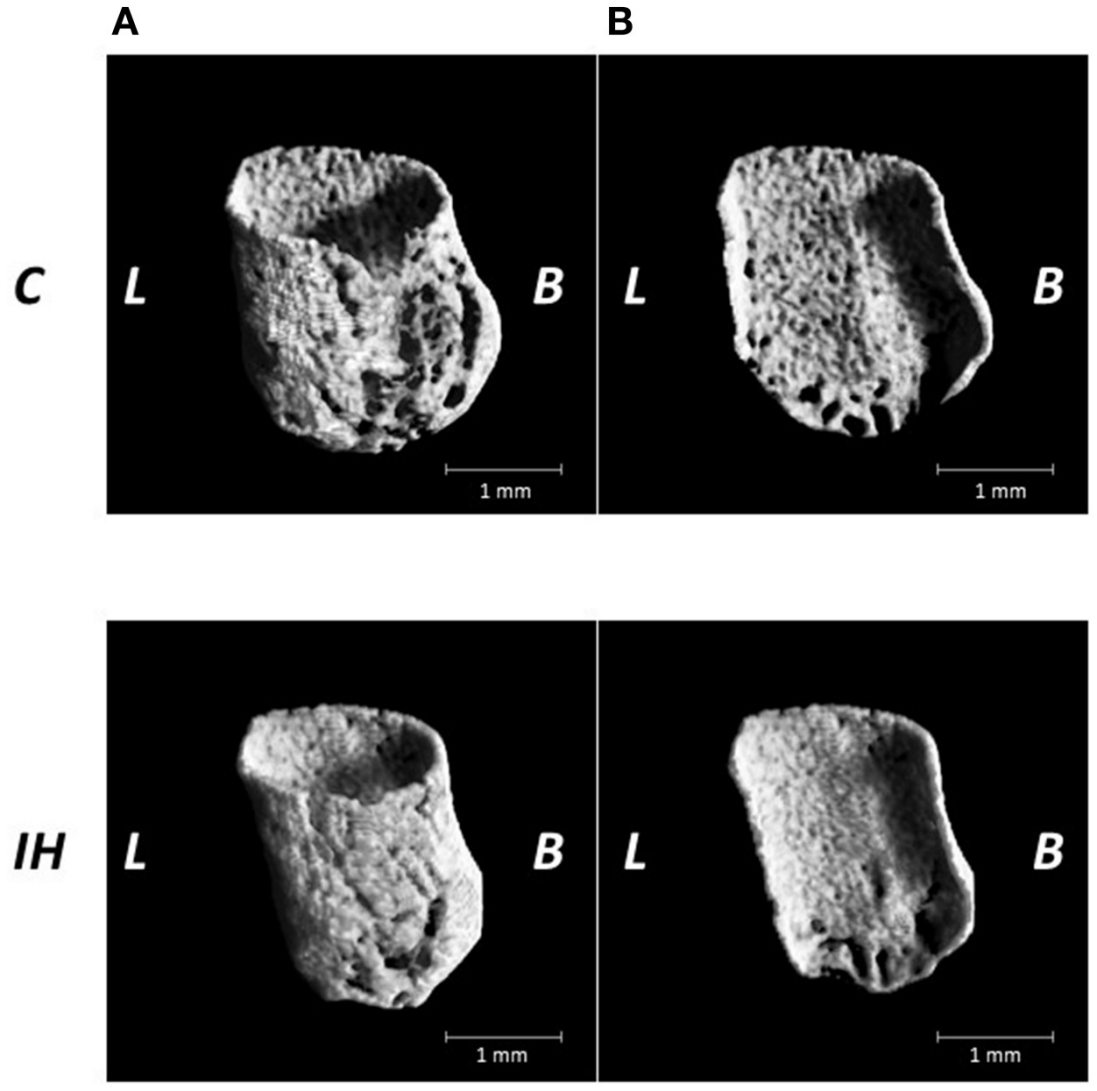
**Microarchitecture of the alveolar bone proper of the distal roots in the mandibular first molar region in the C and IH groups**. Representative micro-CT images of alveolar bone proper in the distal root of mandibular first molar region **(A)** and half of mesial surface **(B)**. Scale bar: 1.0 mm. C, control group; IH, IH group; L, lingual; B, buccal.

**Figure 3:**
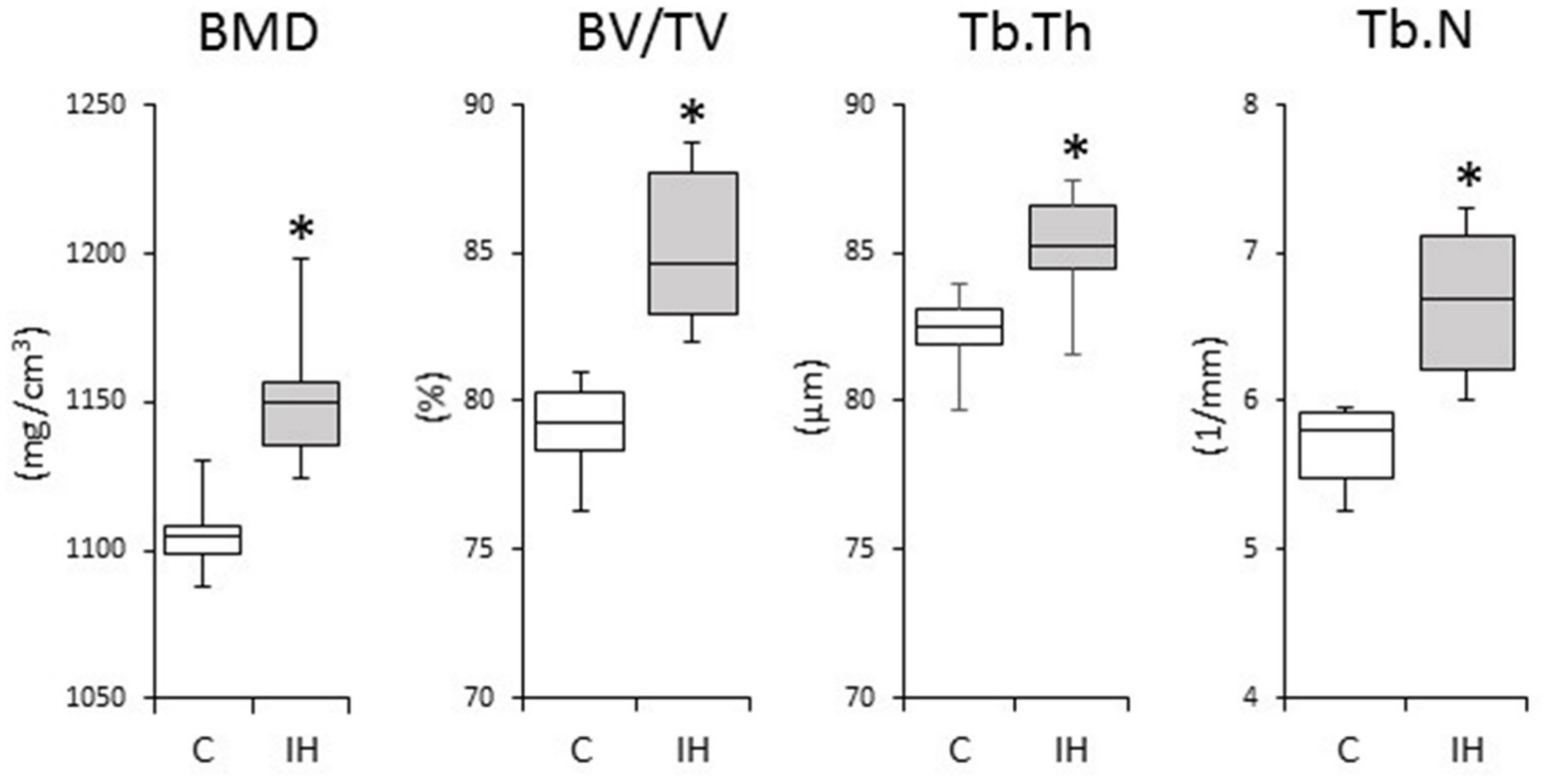
**Comparisons of bone morphology between the C and IH groups by micro-CT analysis**. Cancellous bone of the alveolar bone proper of the distal roots in the mandibular first molar region was compared between the C and IH groups. Box edges represent the upper and lower quantiles, the middle lines in the boxes represent the medians, and the whiskers represent the maxima and minima. BMD, bone mineral density; BV/TV, bone volume/tissue volume; Tb.Th, trabecular thickness; Tb.N, trabecular number. ^*^p < 0.05 by the Mann–Whitney *U*-test.

We investigated changes in the bony microstructure of the alveolar bone proper around the distal roots of mandibular M1 using micro-CT. Micro-CT images of alveolar bone proper around the distal root of mandibular M1 showed higher bone volume (BV) density in the IH rats than in the C rats (Figure 2). In addition, micro-CT analyses demonstrated significant increases in the BMD, BV/TV, Tb.Th, and Tb.N in the distal root alveolar bone proper of IH rats, compared with those of C rats (Figure 3).

### Quantitative real-time PCR

**Figure 4:**
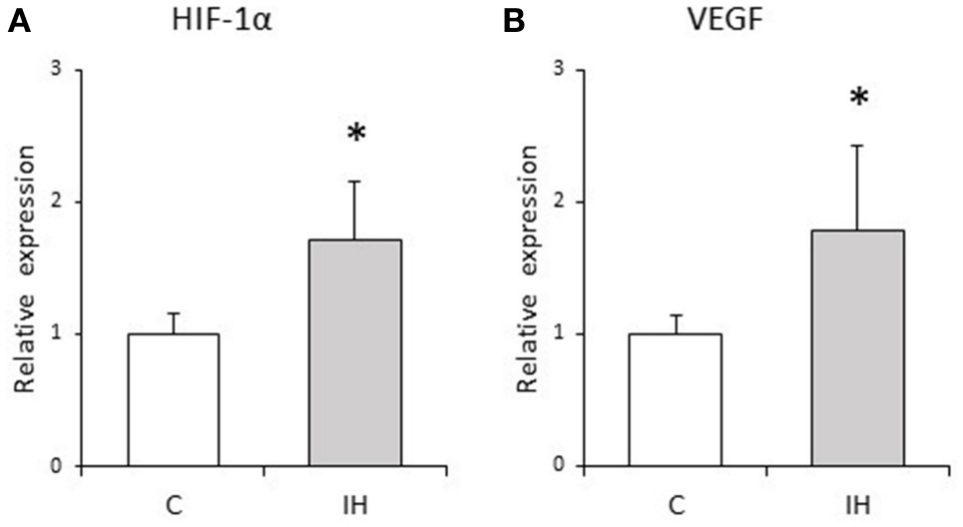
**Relative expression levels of osteogenesis–angiogenesis coupling markers**. Relative HIF-1α **(A)** and VEGF **(B)** expression in the PDL tissues was compared between the C and IH rat groups by RT-qPCR. The mRNA-expression levels measured in C rats were set to a value of 1. Data are shown as the mean ± standard deviation for each group. ^*^p < 0.05 by the Mann–Whitney *U*-test.

**Figure 5:**
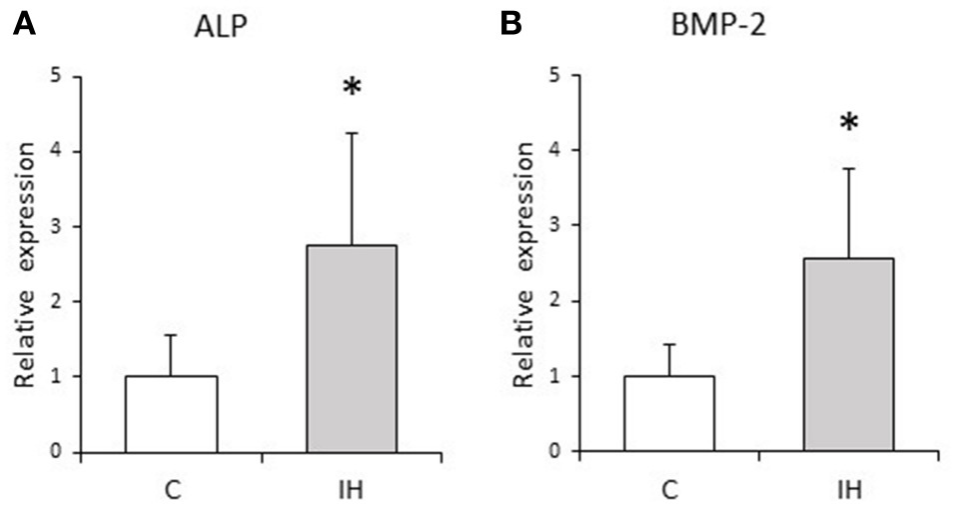
**Relative expression levels of osteogenic markers**. Relative ALP **(A)** and BMP-2 **(B)** expression in the PDL tissues was compared between the C and IH rat groups by RT-qPCR. The mRNA-expression levels measured in C rats were set to a value of 1. Data are shown as the mean ± standard deviation for each group. ^*^p < 0.05 by the Mann–Whitney *U*-test.

**Figure 6:**
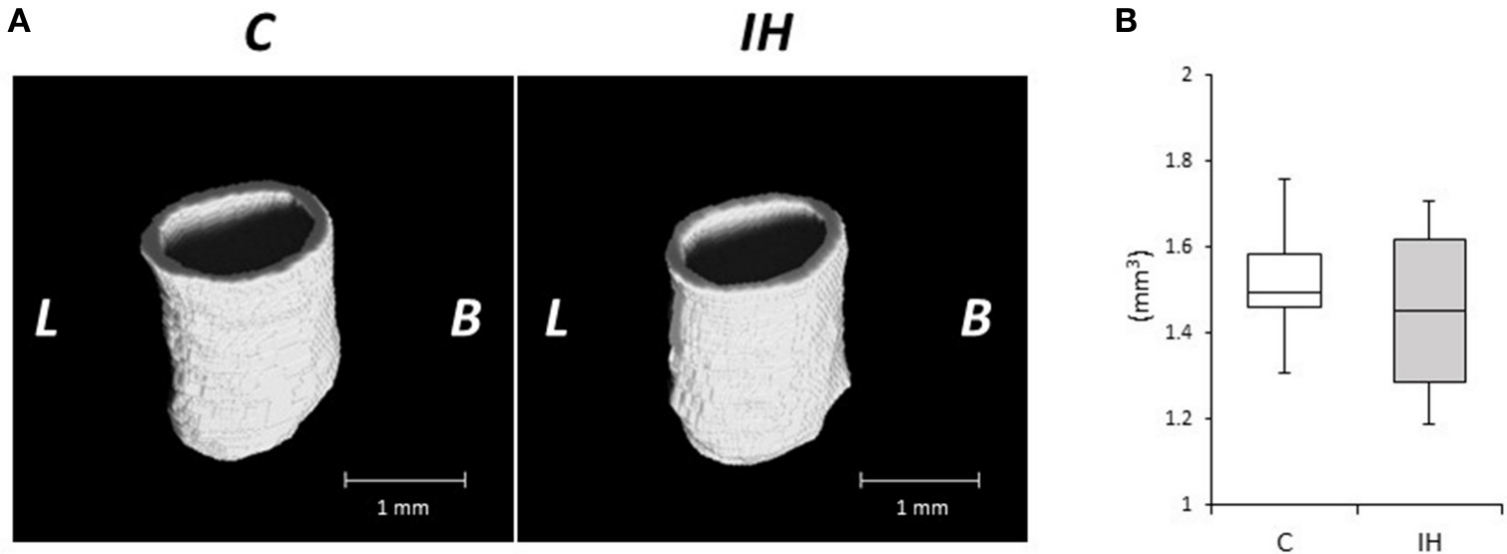
**Micro-CT analysis of the periodontal ligament (PDL) space around the distal roots in the mandibular first molar. (A)** Three-dimensional reconstructed images of the periodontal ligament (PDL) space. Scale bar: 1.0 mm. C, control group; IH, IH group; L, lingual; B, buccal. **(B)** Comparison of tissue volume (TV) of the PDL around the distal root of the mandibular first molar. Box edges represent the upper and lower quantiles with the median values shown by the middle line in each box. The whiskers represent the maxima and minima. There was no significant difference in TV in the PDL between the 2 groups. ^*^p < 0.05 by the Mann–Whitney *U*-test.

We evaluated the relative expression levels of osteogenesis–angiogenesis coupling markers (Figure 4) and osteogenic markers (Figure 5) in PDL tissues by RT-qPCR analysis. IH increased the mRNA levels of HIF-1α and VEGF by 1.71- and 1.97-fold, respectively, in PDL tissues compared with those observed in C rats (Figure 4). Similarly, ALP and BMP-2 mRNA levels were increased 2.75- and 2.57-fold in PDL tissues compared to the corresponding levels in C rats (Figure 5). These changes are not likely to have been mediated by mechanical forces, as IH had no effect on the PDL space surrounding the distal root of the mandibular M1 (Figure 6).

## Discussion

The null hypothesis that no significant differences would occur in the mRNA-expression levels of some factors associated with the HIF pathway in PDL between control rats and IH rats was rejected: IH enhanced osteogenesis in the mandibular M1 region in association with VEGF, ALP, and BMP-2 gene up-regulation in PDL tissues. These molecules are abundantly expressed in osteoblasts and promote angiogenesis in association with osteogenesis via the HIF-1α pathway (Mamalis and Cochran, 2011).

Previously, we reported that morphological changes occur in craniofacial bone after IH exposure in growing rats. IH suppressed development of the nasal cavity and decreased the size of the mandibular and viscerocranial bones, which could have disturbed nasal breathing (Kuma et al., 2014; Oishi et al., 2015). Oxygen is indispensable for enzymatic reactions to promote tissue development, whereas HIF and VEGF play pivotal survival roles under hypoxic conditions (Maes et al., 2012). In the elderly, it was reported that OSA is associated with an increase in BMD, which suggests that IH can stimulate bone remodeling (Sforza et al., 2013). Moreover, activation of the HIF pathway by hypoxia-mimicking agents prevents bone loss in estrogen-deficient mice, but increases BMD and trabecular microarchitecture (Peng et al., 2014). Data from the present study revealed enhanced BMD and bone development in the alveolar bone proper in the mandibular M1 region after IH exposure (Figures 2, 3). The 3 weeks of IH exposure starting at the age of 7 weeks in rats reflects pre-puberal development (Sengupta, 2013). Given that remodeling of inter-radicular alveolar bone, such as alveolar bone proper, in the mandibular M1 region is promoted by signals from PDL tissues (Kaku and Yamauchi, 2014), we considered that IH induced the peri-M1 changes via enhanced coupling in the signaling pathways for osteogenesis and angiogenesis in the PDL.

HIF-1α, a member of the HIF subfamily, is a ubiquitously expressed transcription factor that regulates cellular adaptation under hypoxia (Liu and Simon, 2004). VEGF is transcriptionally activated by HIF and positively regulates angiogenesis (Miyagawa et al., 2009; Wan et al., 2010). Previous findings have indicated that HIF-1α promotes both angiogenesis and osteogenesis via VEGF upregulation in osteoblasts (Wang et al., 2007). In addition, it was reported that VEGF stimulates the differentiation and chemotactic migration of osteoblastic cells (Hankenson et al., 2015), whereas HIFs and VEGF are involved in skeletal development and bone homeostasis (Wan et al., 2010; Maes et al., 2012). IH stimulates vessel network formation and VEGF production in a highly correlated fashion (Ehsan and George, 2015). The vascular system not only supplies nutrients and oxygen to developing bones, but also delivers critical signals that stimulate mesenchymal cell differentiation toward an osteogenic phenotype, whereas HIF triggers the initiation and promotion of angiogenic-osteogenic cascade events (Mamalis and Cochran, 2011). It was also shown that hypoxia promotes VEGF production in PDL cells (Motohira et al., 2007) and that the PDL plays an important role in tooth eruption (Kaku and Yamauchi, 2014). Consistent with these findings, we found increased HIF-1α and VEGF mRNA expression in peri-M1 PDL tissues after IH exposure (Figure 4), and the lack of change observed in the PDL space (Figure 6) suggests that, not mechanical force, but chemical stimulation related to HIF-1α from PDL tissues induced peri-M1 osteogenesis. Collectively, our findings indicate that HIF-1α mediated VEGF induction in PDL tissues and that VEGF induced peri-M1 osteogenesis.

We evaluated osteogenesis by studying ALP and BMP-2 expression. Given that ALP is a well-known indicator of osteoblastic differentiation, ALP expression indicates the presence of osteoblasts and osteogenic activity (Kuru et al., 1999; Nettelhoff et al., 2016). BMP-2, a member of the transforming growth factor beta superfamily, induces osteogenesis from PDL cells and the regeneration of alveolar bone by promoting osteoblastic differentiation (Selvig et al., 2002). We found significantly higher ALP and BMP-2 expression levels in PDL tissues in rats after IH exposure (Figure 5), which are thought to induce osteogenesis in the alveolar bone proper.

Cephalometric analysis indicated small mandibular sizes and posterior displacements in patients with OSA (Rivlin et al., 1984). Children with OSA present with increased over jet, reduced over bite, narrowed upper dental arches, and shorter lower dental arches (Pirilä-Parkkinen et al., 2009). Oral appliances and mandibular-advancement devices have been used to treat abnormal craniofacial development, such as morphological changes and intermaxillary relations in patients with OSA (Almeida et al., 2006; Hou et al., 2006). It has been suggested that a priori changes in craniofacial bones induce pathogenesis in patients with OSA.

Data from previous reports have indicated that the morphologies of mandibular and dental changes play primary roles in the development of OSA pathophysiology, whereas the present data indicated that the changes of molecular mechanism about PDL tissues in IH rat model. The osteogenesis–angiogenesis coupling phenomenon with HIF-1α and VEGF was involved in the increased BMD observed in developing alveolar bone under IH. Collectively, our data demonstrated for the first time that short periods of IH exposure can enhance peri-M1 abnormally osteogenesis via the HIF-1α-VEGF pathway in growing rats. Similar mechanisms may occur in the craniofacial bones in young children under IH exposure. Our results suggested the necessity of early treatment in children with OSA to maintain normal bone growth. Furthermore, we also demonstrated the usefulness of this IH model in studying the molecular mechanism underlying the morphological changes occurring in craniofacial bones in children with OSA. Although IH has been strongly implicated in OSA pathogenesis, OSA is associated with multifactorial pathogenesis, such as hypercapnia, intrathoracic negative pressure, and sympathetic overactivation (Fletcher, 2001). IH exposure as a single pathogenesis of OSA, a limitation of this study, is that the morphological changes observed in rats may differ somewhat from those that occur in children with OSA; thus, further morphological studies on pediatric OSA are required. Further study is also expected under different experimental settings, involving differences in IH exposure and age.

In conclusion, we demonstrated that IH increases BMD in alveolar bone proper around the roots of the first mandibular molar. Our data suggest the involvement of an osteogenesis–angiogenesis coupling phenomenon with HIF-1α and VEGF in PDL tissues. IH was found to significantly increase BMD and alter bone microstructure, potential risk factors for homeostasis disturbance in alveolar bone proper in growing IH rats. The expression levels of HIF-1α, VEGF, ALP, and BMP-2 transcripts were up-regulated in PDL tissues subjected to IH exposure for 3 weeks. Although the signaling pathway underlying IH-induced changes in the bony microstructure is not yet fully elucidated, these findings improve our current understanding of the molecular mechanisms underlying the impacts of IH on bone homeostasis.

## Author contributions

SO, YS, JH, YK, HM, HN, RU, SK, NS, JS, KY, and TO conceived of and designed the study; SO performed the experiments; SO, YS, JH, and TO analyzed the data; SO, YS, JH, YK, HM, HN, RU, SK, NS, JS, KY, and TO interpreted the results of the experiments; SO and YS prepared the figures; SO drafted the manuscript; YS, JH, JS, and TO edited the manuscript; All authors approved the final version of the manuscript.

## Funding

This study was financially supported in part by Grants-in-Aid for Scientific Research (25463170, 26463089) from the Japanese Ministry of Education, Culture, Sports, Science, and Technology (KAKENHI).

### Conflict of interest statement

The authors declare that the research was conducted in the absence of any commercial or financial relationships that could be construed as a potential conflict of interest.
